# Ethnic group and survival from childhood cancer: report from the UK Children's Cancer Study Group

**DOI:** 10.1054/bjoc.1999.1101

**Published:** 2000-03-06

**Authors:** C A Stiller, K J Bunch, I J Lewis

**Affiliations:** Childhood Cancer Research Group, Department of Paediatrics, University of Oxford, 57 Woodstock Road, Oxford, OX2 6HJ, UK; Department of Paediatric Oncology, St James's University Hospital, Leeds, LS9 7TF, UK

**Keywords:** child, survival, ethnic group, leukaemia

## Abstract

Survival following cancer was analysed in relation to ethnic group among children diagnosed in Britain during 1981–1996 and treated at paediatric oncology centres by members of the UK Children's Cancer Study Group. Survival was analysed for 11 diagnostic groups: acute lymphoblastic leukaemia (ALL), acute non-lymphocytic leukaemia, Hodgkin's disease, non-Hodgkin's lymphoma, astrocytoma, primitive neuroectodermal tumour, neuroblastoma, Wilms' tumour, osteosarcoma, Ewing's sarcoma and rhabdomyosarcoma. There were no significant differences in survival between White and non-White children over the study period as a whole. Among children with ALL, however, the relative risk of death allowing for period of diagnosis, age and white blood count was 1.25 for those of South Asian ethnic origin compared with Whites (*P* = 0.057). © 2000 Cancer Research Campaign


British Journal of Cancer (2000) **82**(7), 1339–1343
© 2000 Cancer Research Campaign
DOI: 10.1054/ bjoc.1999.1101, available online at http://www.idealibrary.com on IDEAL®

### Ethnic group and survival from childhood cancer: report from the UK Children's Cancer Study Group

**CA Stiller[1], KJ Bunch[1] and IJ Lewis[2]**

[1]Childhood Cancer Research Group, Department of Paediatrics, University of Oxford, 57 Woodstock Road, Oxford OX2 6HJ, UK; [2]Department of Paediatric Oncology, St James's University Hospital, Leeds LS9 7TF, UK

**Summary** Survival following cancer was analysed in relation to ethnic group among children diagnosed in Britain during 1981–1996 and treated at paediatric oncology centres by members of the UK Children's Cancer Study Group. Survival was analysed for 11 diagnostic groups: acute lymphoblastic leukaemia (ALL), acute non-lymphocytic leukaemia, Hodgkin's disease, non-Hodgkin's lymphoma, astrocytoma, primitive neuroectodermal tumour, neuroblastoma, Wilms' tumour, osteosarcoma, Ewing's sarcoma and rhabdomyosarcoma. There were no significant differences in survival between White and non-White children over the study period as a whole. Among children with ALL, however, the relative risk of death allowing for period of diagnosis, age and white blood count was 1.25 for those of South Asian ethnic origin compared with Whites ($P$ = 0.057). © 2000 Cancer Research Campaign

**Keywords:** child; survival; ethnic group; leukaemia

An increasing proportion of the childhood population of the UK are members of diverse ethnic groups. Patterns of childhood cancer incidence vary between the principal ethnic groups in Britain (Stiller et al, 1991; Powell et al, 1994). In particular, children of South Asian origin have a raised incidence of lymphoma and a deficit of Wilms' tumour and soft-tissue sarcoma compared with White children, while among Black children Wilms' tumour is relatively common but Ewing's sarcoma is virtually never seen. There is, however, no apparent ethnic variation in childhood leukaemia incidence in Britain.

Little has been published on the survival rates from cancer of British children from ethnic minorities. In one small study of patients entered in national trials for acute lymphoblastic leukaemia (ALL), the survival rate among children of Asian origin was lower than among White children with similar clinical prognostic features (Oakhill and Mann, 1983). In the US, survival rates for Black children with leukaemia have been found to be lower than those for White children in several studies (Novakovic, 1994; Pui et al, 1995). Survival rates for childhood cancer in developing countries are seldom available but in general they appear to be substantially lower than in Western industrialized countries. The relative contributions to differences in survival rates of biological variations in types of childhood cancer between populations and of differing levels of availability of medical care are unknown.

In this paper we present the results of the first large-scale, national analysis of survival from childhood cancer in Britain in relation to ethnic group.

*Received 23 June 1999*
*Revised 11 October 1999*
*Accepted 12 October 1999*

*Correspondence to:* CA Stiller

### PATIENTS AND METHODS

Survival rates by ethnic group were analysed for children with cancer who were under the care of members of the United Kingdom Children's Cancer Study Group (UKCCSG) at paediatric oncology centres throughout Great Britain. Since the formation of the UKCCSG in 1977, all children with cancer who were patients at these centres have been included in the UKCCSG register. The data include the age and sex of the children and details of the diagnosis. Since 1981, information on ethnic group has also been collected. This is classified as White, South Asian (including Indian, Pakistani, Bangladeshi and East African Asian), Black (African or Caribbean), East Asian (including Chinese and Japanese), Middle Eastern (including Arab) and Other (including combinations of the above).

Follow-up is obtained in several ways. Registrations are linked with the population-based National Registry of Childhood Tumours (NRCT), which receives death certificates for all persons who die in Britain under the age of 20 with a neoplasm coded as the underlying cause. About 5 years from diagnosis, children in the NRCT for whom no death certificate has been received are flagged in the National Health Service Central Registers (NHSCR) who then notify any further deaths together with embarkations resulting in loss to follow-up. In this study, flagged patients for whom no death or embarkation had been notified were assumed to be still alive on 31 August 1998. Annual follow-up data for children who are not already known to have died and have not been flagged are obtained from the databases of the clinical trials and studies of the UKCCSG and by direct enquiry to the consultants responsible for their care.

The UKCCSG register included 15 829 children who were diagnosed with a malignant neoplasm or a non-malignant intracranial or intraspinal tumour during 1981–1996. After excluding 484 children of unknown ethnic group, and 2734 in diagnostic groups with fewer than five deaths expected among non-White children, 12 611 children in 11 diagnostic groups remained for analysis.

**Table 1** Numbers of children included in the survival analyses by ethnic group and diagnostic group in the International Classification of Childhood Cancer

| | White | All other[b] | South Asian | Black |
|---|---|---|---|---|
| ALL | 4099 | 442 | 281 | 46 |
| ANLL | 805 | 85 | 41 | 10 |
| Hodgkin's disease | 555 | 98 | 62 | 18 |
| NHL[a] | 916 | 100 | 58 | 11 |
| Astrocytoma | 938 | 68 | 37 | 10 |
| PNET | 636 | 67 | 41 | 10 |
| Neuroblastoma | 1126 | 133 | 68 | 19 |
| Wilms' tumour | 998 | 101 | 39 | 33 |
| Osteosarcoma | 278 | 26 | 14 | 6 |
| Ewing's sarcoma | 260 | 26 | 17 | 1 |
| Rhabdomyosarcoma | 781 | 73 | 26 | 13 |

[a]Including Burkitt's and unspecified. [b]Including mixed.

Table 1 shows the numbers of children in the series, classified by ethnic group and by diagnostic group in the International Classification of Childhood Cancer (Kramarova and Stiller, 1996).

Survival rates were calculated by standard actuarial methods and differences between survival curves were tested by log-rank tests. Two sets of analyses were performed. The first compared White children and those of all other ethnic groups combined. The second compared survival of children from individual minority ethnic groups with that of White children. In both sets of analyses, combinations of diagnostic group and ethnic group for which fewer than five deaths were expected were excluded. As survival has improved substantially for most types of childhood cancer since 1980 (Stiller, 1994) and the distribution of ethnic group among children with cancer has also changed, calendar period of diagnosis was allowed for as a stratified variable, grouped as 1981–1986, 1987–1991 and 1992–1996. Survival from ALL is markedly dependent upon age at diagnosis and white cell count; age, at least, varies between ethnic groups. Accordingly, further analyses were done in which these two factors were allowed for as stratified variables, with age grouped as 0, 1–4 and 5–14 years and white cell count as under and over $20 \times 10^9$ $l^{-1}$ or unknown. Age is also an important prognostic factor for neuroblastoma and was again allowed for as a stratified variable, this time grouped as 0, 1–2 and 3–14 years.

### RESULTS

Table 2 shows the actuarial 5-year survival rates for White children and those of all other ethnic groups combined in each of the 11 diagnostic groups, separately for patients diagnosed during 1981–1986, 1987–1991 and 1992–1996. Observed and expected numbers of deaths and the results of a log-rank test are also presented; these are for the whole period 1981–1996, but allowing for period of diagnosis. No diagnostic group showed a significant difference in survival between White and non-White children overall. Non-White children had a lower survival rate than White children with ALL – relative risk (RR) of death in minority group = 1.14; the difference in survival became more marked beyond 5 years after diagnosis (Figure 1). Among children diagnosed during 1987–1991, the non-White group had significantly worse survival (RR = 1.42, $P$ = 0.023), and allowing for age and white blood count made little difference to this result (RR = 1.39, $P$ = 0.029). Survival from astrocytoma was significantly worse among non-White children diagnosed during 1981–1986 (RR = 3.14, $P$ = 0.0022). Non-White children had a significantly higher survival

**Table 2** Survival of White and non-White children by diagnostic group, 1981–1996

| | | Five-year % survival (SE) | | | | | 2-sided |
|---|---|---|---|---|---|---|---|
| | | 1981–1986 | 1987–1991 | 1992–1996 | O | E | *P*-value |
| ALL | White | 71 (1.3) | 76 (1.2)* | 82 (1.3) | 1068 | 1081.8 | 0.17 |
| | Other | 74 (4.0) | 70 (4.0) | 84 (3.0) | 122 | 108.2 | |
| ANLL | White | 30 (2.8) | 48 (3.1) | 56 (3.3) | 446 | 442.8 | 0.63 |
| | Other | 48 (10.9) | 54 (10.2) | 54 (8.1) | 43 | 46.2 | |
| Hodgkin's disease | White | 93 (1.8) | 94 (2.1) | 97 (1.3) | 44 | 43.8 | 0.98 |
| | Other | 93 (4.6) | 96 (3.5) | 97 (2.5) | 7 | 7.2 | |
| NHL | White | 68 (2.7) | 76 (2.5) | 79 (2.4) | 244 | 247.0 | 0.52 |
| | Other | 76 (8.5) | 72 (7.5) | 59 (14.4) | 29 | 26.0 | |
| Astrocytoma | White | 69 (3.2)* | 65 (3.0) | 70 (2.5) | 298 | 304.1 | 0.16 |
| | Other | 25 (12.5) | 69 (11.6) | 72 (7.2) | 26 | 19.9 | |
| PNET | White | 53 (3.8) | 46 (3.5) | 50 (3.6) | 338 | 337.1 | 0.90 |
| | Other | 47 (12.9) | 35 (11.6) | 58 (8.6) | 35 | 35.9 | |
| Neuroblastoma | White | 42 (2.7) | 42 (2.4) | 55 (3.1) | 603 | 607.9 | 0.52 |
| | Other | 46 (7.8) | 40 (7.3) | 24 (17.7) | 73 | 68.1 | |
| Wilms' tumour | White | 80 (2.3) | 81 (2.1) | 77 (2.5) | 201 | 197.5 | 0.42 |
| | Other | 84 (6.4) | 88 (6.3) | 80 (6.4) | 17 | 20.5 | |
| Osteosarcoma | White | 51 (5.4) | 56 (5.7) | 56 (5.9) | 125 | 126.7 | 0.55 |
| | Other | 50 (15.8) | 100 | 58 (13.1) | 12 | 10.3 | |
| Ewing's sarcoma | White | 44 (4.9)* | 69 (5.0) | 58 (8.6) | 114 | 109.5 | 0.14 |
| | Other | 83 (15.2) | 78 (13.9) | 70 (14.7) | 7 | 11.5 | |
| Rhabdomyosarcoma | White | 57 (3.1) | 61 (3.1) | 67 (3.2) | 309 | 311.9 | 0.55 |
| | Other | 65 (11.5) | 48 (9.3) | 62 (10.3) | 31 | 28.1 | |

The expected numbers of deaths and log-rank tests are based on stratified analyses allowing for calendar period of diagnosis O = observed deaths, E = expected deaths. [a] $P < 0.05$ on log-rank test for calendar period.

 *© 2000 Cancer Research Campaign*

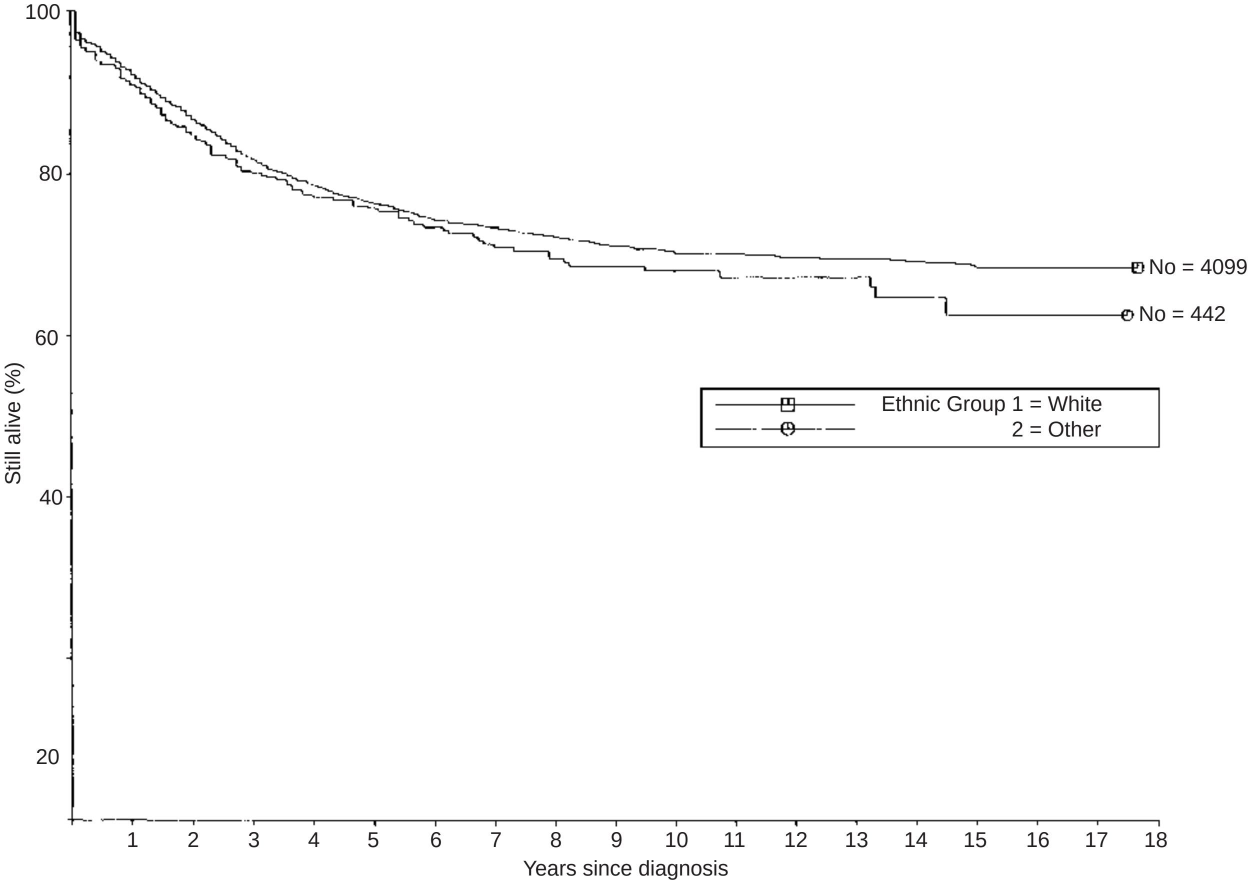

**Figure 1** Actuarial survival of White and non-White children with acute lymphoblastic leukaemia, 1981–1996

**Table 3** Survival of children by diagnostic group and ethnic group, 1981–1986

| | White | South Asian | | Black | | Middle Eastern | | East Asian | |
|---|---|---|---|---|---|---|---|---|---|
| | 5 year % survival (SE) | 5-year % survival (SE) | 2*P* | 5-year % survival (SE) | 2*P* | 5-year % survival (SE) | 2*P* | 5-year % survival (SE) | 2*P* |
| ALL | 76 (0.7) | 75 (2.7) | 0.070 | 81 (6.2) | 0.79 | 62 (10.1) | 0.33 | 71 (9.4) | 0.45 |
| ANLL | 45 (1.8) | 49 (7.8) | 0.90 | 56 (16.6) | 0.47 | | | | |
| NHL | 74 (1.5) | 73 (6.0) | 0.99 | | | | | | |
| Astrocytoma | 69 (1.6) | 59 (8.3) | 0.15 | | | | | | |
| PNET | 49 (2.1) | 43 (8.5) | 0.91 | 56 (16.5) | 0.90 | | | | |
| Neuroblastoma | 46 (1.5) | 46 (6.4) | 0.69 | 32 (10.7) | 0.013 | | | | |
| Wilms' tumour | 80 (1.3) | 74 (7.2) | 0.40 | 88 (5.8) | 0.48 | | | | |
| Osteosarcoma | 54 (3.2) | 63 (13.1) | 0.69 | | | | | | |
| Ewing's sarcoma | 56 (3.2) | 76 (10.5) | 0.29 | | | | | | |
| Rhabdomyosarcoma | 61 (1.8) | 64 (9.7) | 0.69 | 58 (14.4) | 0.99 | | | | |

The log-rank tests are based on comparisons with White children, allowing for calendar period grouped as 1981–1986, 1987–1991, 1992–1996.

rate than White children with Ewing's sarcoma diagnosed during 1981–1986 (RR = 0.19, $P$ = 0.033). For most other diagnostic groups there was little evidence of any variation in survival between White and non-White children.

Table 3 shows the results of analysing survival rates for children from individual minority ethnic groups compared with White children. The poorer survival of South Asian children (RR = 1.23) with ALL was of borderline statistical significance; again the

© 2000 Cancer Research Campaign 

difference was almost entirely in survival beyond 5 years after diagnosis. In a further analysis allowing simultaneously for the effects of age and white blood count in addition to calendar period, there was little change in the results (RR = 1.25, $P = 0.057$).

Black children with neuroblastoma had a lower survival rate than White children with this tumour (RR = 2.12) and the difference was highly significant. This was partly accounted for by the fact that only 2/19 (11%) Black children were aged under 1 year at diagnosis, compared with 322/1126 (29%) of White children but, when age was allowed for, the poorer survival of Black children was still significant (RR = 1.79, $P = 0.047$).

### DISCUSSION

A sizeable proportion of the population of Britain is of minority ethnic origin, yet there is a surprising lack of published information on survival from cancer by ethnic group in Britain (Selby, 1996). Cancer mortality in immigrants to Britain has been studied (Grulich et al, 1992; Swerdlow et al, 1995) but it is unclear how much the variations are due to differences in incidence rather than survival.

Most previous studies of cancer survival in relation to ethnic group have been carried out in the USA. For children, these have usually compared the results for ALL, the commonest childhood cancer, between White and Black patients. The present study is the first large-scale investigation of survival from cancer by ethnic group at any age in Britain, and the first multi-centre study anywhere in the world to consider a wide range of childhood cancers in addition to ALL.

There was little evidence of any ethnic differences in survival for most of the diagnostic groups studied. The number of statistically significant results was no greater than would be expected to occur by chance among the number of comparisons made.

US cancer registration data consistently show differing patterns of childhood ALL incidence among Blacks and Whites, with the early childhood peak of good-prognosis disease being much less marked in Black children (Parkin et al, 1988). Black children with ALL treated at St Jude Children's Research Hospital during 1962–1983 had a much lower survival rate than other, predominantly White, children but during 1984–1992 there was no difference in survival rates (Pui et al, 1995); these results were little changed by adjusting for age and white blood count. In contrast, population-based data from the SEER Program suggested that the gap between Black and White children's survival rates widened between 1978–1982 and 1983–1987, with survival actually deteriorating for blacks in the more recent period (Novakovic, 1994); the contribution of differences in the age distribution of patients between the ethnic groups was not assessed. It seems unlikely that the discrepancy between the two studies can be explained by a concentration of Black children at non-specialist hospitals with a very low survival rate, as virtually all American children with ALL in recent years are believed to have been treated at centres affiliated to one of the major cooperative trials groups (Ross et al, 1996).

Less has been published on ethnic differences in survival from other childhood cancers. The St Jude study found relatively little evidence for differences in survival between ethnic groups for cancers other than ALL. The lack of significant results may simply be a consequence of the smaller numbers of cases available for analysis, though in both studies there was little difference between ethnic groups in the observed survival rates for several types of cancer. The lower survival rate we found among Black children with neuroblastoma has not previously been reported. The higher survival rate among ethnic minority children (predominantly South Asian) with Ewing's sarcoma is also a new finding.

The ethnic group classification used has limitations. The largest non-White category, South Asian, includes people from diverse socioeconomic, cultural and religious backgrounds. They, or their parents, could have been born anywhere in the Indian sub-continent, East Africa, or Britain. Yet even in this group there were under 300 children with ALL, by far the commonest cancer in this study. Use of any more specific classification would have substantially reduced the numbers of children in each group and would also almost certainly have increased the amount of misclassification.

Information on the health of ethnic minority children in Britain is sparse. The available evidence suggests that overall morbidity and mortality are higher than in White children (Raleigh and Balarajan, 1995). Ethnic origin and socio-economic status are confounded. In a recent study, the use of medical services by children and young people did not vary with socioeconomic status but members of the principal ethnic minority groups were less likely than White children and young people to use hospital inpatient and outpatient services, which could imply that they receive a poorer quality of health care (Cooper et al, 1998). The present study indicates, however, that for cancers that are a principal cause of death at age 1–14, the survival rate is relatively uniform across ethnic groups. The main exception is the possibly lower long-term survival of ethnic minority children with ALL, particularly those of South Asian origin. Possible explanations might include ethnic variations in drug metabolism, susceptibility to infection or compliance with therapy. The previously reported poorer prognosis of Asian children who were included in Medical Research Council ALL trials was almost entirely attributable to a very much increased rate of death in remission (Oakhill and Mann, 1983). Remission status was not recorded in our data.

Survival from ALL among ethnic minority children should continue to be monitored. If they appear to suffer persistently lower survival rates, more detailed research will be required to establish why this should be so and hence to indicate possible means of eliminating the difference.

### ACKNOWLEDGEMENTS

We are grateful to members of the UK Children's Cancer Study Group and the Office for National Statistics and the Registrar General for Scotland for providing information on cases of childhood cancer and also to the NHS Central Registers in Southport and Edinburgh for notifications of deaths and for flagging survivors. We thank the many people who have worked on the National Registry of Childhood Tumours, particularly Ms AM Bayne, Dr EM Eatock, Mr MJ Loach, Mrs P Brownbill, Mrs J Williams, Miss A Berry and Miss A Sabin. We thank Mrs EM Roberts for secretarial assistance. The Childhood Cancer Research Group is supported by the Department of Health and the Scottish Home and Health Department. The UKCCSG is supported by the Cancer Research Campaign.

 © 2000 Cancer Research Campaign

### REFERENCES

Cooper H, Smaje C and Arber S (1998) Use of health services by children and young people according to ethnicity and social class: secondary analysis of a national survey. *Br Med J* **317:** 1047–1051

Grulich AE, Swerdlow AJ, Head J and Marmot MG (1992) Cancer mortality in African and Caribbean migrants to England and Wales. *Br J Cancer* **66:** 905–911

Kramarova E and Stiller CA (1996) The international classification of childhood cancer. *Int J Cancer* **68:** 759–765

Novakovic B (1994) US childhood cancer survival, 1973–87. *Med Pediatr Oncol* **23**: 480–486

Oakhill A and Mann JR (1983) Poor prognosis of acute lymphoblastic leukaemia in Asian children living in the United Kingdom. *Br Med J* **286**: 839–841

Parkin DM, Stiller CA, Draper GJ and Bieber CA (1988) The international incidence of childhood cancer. *Int J Cancer* **42**: 511–520

Powell JE, Parkes SE, Cameron AH and Mann JR (1994) Is the risk of cancer increased in Asians living in the UK? *Arch Dis Child* **71**: 398–403

Pui C-H, Boyett JM, Hancock ML, Pratt DB, Meyer WH and Crist WM (1995) Outcome of treatment for childhood cancer in black as compared with white children. The St Jude Children’s Research Hospital experience, 1962 through 1992. *JAMA* **273**: 633–637

Raleigh VS and Balarajan R (1985) The health of infants and children among ethnic minorities. In: *The Health of our Children*, Decennial Supplement, Series DS no. 11, Botting B (ed), pp. 82–94. HMSO: London

Ross JA, Severson RK, Pollock BH and Robison LL (1996) Childhood cancer in the United States. A geographical analysis of cases from the pediatric co-operative clinical trials groups. *Cancer* **77**: 201–207

Selby P (1996) Cancer clinical outcomes for minority ethnic groups. *Br J Cancer* **74**: S54–S60

Stiller CA (1994) Population-based survival rates for childhood cancer in Britain, 1980–91. *Br Med J* **389**: 1612–1616

Stiller CA, McKinney PA, Bunch KJ, Bailey CC and Lewis IJ (1991) Childhood cancer and ethnic group in Britain: a United Kingdom Children’s Cancer Study Group (UKCCSG) study. *Br J Cancer* **64**: 543–548

Swerdlow AJ, Marmot MG, Grulich AE and Head J (1995) Cancer mortality in Indian and British ethnic immigrants from the Indian subcontinent to England and Wales. *Br J Cancer* **72**: 1312–1319

© 2000 Cancer Research Campaign
